# Attention and executive control in varsity athletes engaging in strategic and static sports

**DOI:** 10.1371/journal.pone.0266933

**Published:** 2022-04-22

**Authors:** Alma Rahimi, Samantha D. Roberts, Joseph R. Baker, Magdalena Wojtowicz

**Affiliations:** 1 Department of Psychology, York University, Toronto, Ontario, Canada; 2 School of Kinesiology, York University, Toronto, Ontario, Canada; Ghent University, BELGIUM

## Abstract

Examining non-sport-related cognitive tasks of attention and executive control in skilled athletes may provide insight into the acquisition of highly specific skills developed in experts as well as help identify successful performance in sport. Through a cross-sectional design, this study examined performance on aspects of attention and executive control among varsity athletes playing soccer (strategic sport) or track & field (static sport) using a computerized test of attention and executive control. Ninety-seven university athletes participating in soccer (*n* = 50) or track and field (*n* = 47) were included in the study. Domains of attention and executive control were examined using the Attention Network Test-Interactions (ANT-I). Mean reaction time (RT) and intra-individual variability (IIV) were compared between groups as measures of performance speed and performance stability respectively. Soccer players demonstrated overall faster RTs (*p* = 0.0499; ηp2 = .04) and higher response accuracy (*p* = .021, *d* = .48) on the ANT-I compared to track and field athletes. Faster RTs were observed for soccer players when presented with an alerting tone (*p* = .029, *d* = .45), valid orienting cue (*p* = .019, *d* = .49) and incongruent flanker (*p* = .031, *d* = .45). No significant group differences were observed in IIV (*p* = .083, *d* = .36). Athletes engaging in strategic sports (i.e., soccer) demonstrated faster performance under test conditions that required higher vigilance and conflict resolution. These findings suggest that engagement in strategic sports is associated with enhanced performance on non-sport-related cognitive tasks of attention and executive control.

## Introduction

The beneficial effects of exercise on cognitive function across the lifespan are well-documented [1–3]. Individuals who engage in regular physical activity have been found to demonstrate more accurate and faster performance on cognitive measures that require relational memory, aspects of attention, as well as executive function [4, 5]. Executive function is a set of cognitive processes and mental skills that depend on the harmonious interaction between working memory, mental flexibility, and self-control—three types of highly interdependent brain functions that enable individuals to plan, monitor, and achieve their goals [6]. Competitive sports training, which requires high physical and motor fitness, has been associated with greater task-related neural network efficiency [7]. Elite athletes who engage in sports that require high levels of structure (i.e., abiding by a complex set of rules) and coordination (i.e., adapting to variable game situations) have been found to demonstrate superior performance on tasks requiring decision making, perception, and visual searching capacity [8–10]. The perceptual skills that distinguish sporting experts from less-skilled peers in time-constrained decision-making sports seem not only highly specific to the demands of the task but also to performers’ prior practice experiences [11]. Although this suggests a key role for practice in explaining differences between performers, the potential value of general cognitive capacities has re-emerged in discussions of skilled athletic performance (e.g., the value of multiple object tracking) [12]. While the precise role of these general cognitive capacities is not known, it is plausible that increased general attention skill underpins the acquisition of highly specific skills developed in experts and/or influence an individual’s choice of activity during early development (e.g., those with higher capacity will be drawn to sports emphasizing this quality). Examining these general skills across different types of activities would extend our understanding of ‘where’ these skills are valuable, a meaningful first step to understanding ‘why’.

In their meta-analysis of cognition and sport expertise, Voss, Kramer, Basak, Prakash and Roberts [13] used a simple approach that categorized sports into three types: static, interceptive and strategic. Static sports are self-paced, closed-skill, and require independence and consistency (e.g., track and field, swimming). Interceptive sports are externally paced, open-skill, and involve a high degree of body-object coordination (e.g., tennis, badminton). Finally, strategic sports are externally paced, open-skill, and require simultaneous information processing and effective attentional allocation (e.g., soccer, volleyball). Studies have reported differences in performance on aspects of visuomotor speed, visual attention, and executive function associated with the type of sport played [14–22]. For example, athletes who engage in strategic sports have been found to perform better on tasks requiring visual attention and motor inhibition compared to those in interceptive sports [19], as well as better inhibitory control [15, 18] and stability of performance than athletes in static sports [23]. These findings support the cognitive skill transfer hypothesis which posits that skills acquired through training in one cognitive task may improve performance on a related but untrained task [13, 24–27]. In contrast, other studies have not found significant differences in cognitive performance across sport types [28, 29] or have found evidence of superior cognitive performance—such as response inhibition—in static, self-paced sports (e.g., swimming) at a varsity level [30].

To date, research examining cognitive profiles of athletes participating in strategic sports suggest that these athletes may demonstrate better performance on aspects of cognitive functioning related to their training and sport demands [15, 18, 19, 23]. However, studies that have investigated performance variability in attention and executive control measures in these groups are limited [15]. Intra-individual variability (IIV) is defined as the variability of performance across occasions [31]. Those who perform well on cognitive tasks tend to show less variability and more stability than those who perform poorly [32–34]. Therefore, IIV in performance across a set of trials provides unique information about the stability of an individual’s performance, their ability to maintain focus, and may reflect neural integrity of frontal regions [35–38].

Furthermore, the presence of potential confounding factors, such as level of education and concussion history, as well as the use of mixed sport samples in the current literature, make it challenging to appropriately assess the associations between participation in strategic sports and cognitive functioning. The present study examined pre-season performance on measures of speed, accuracy, and stability on a task of attention and executive control (i.e., Attention Network Test-Interactions) [39] in a sample of healthy varsity soccer (i.e., strategic sport) and track and field (i.e., static sport) athletes, comparable on age, sex, education, and level of play. The Attention Network Test (ANT) was originally developed by Fan and colleagues [40] and later modified as the Attention Network Test-Interactions (ANT-I) by Callejas and colleagues [39] to measure attention networks postulated by Posner and Petersen [41] and their interactions in the context of a single task. Since its release, the ANT and its modifications, including the ANT-I, have been widely adopted in various studies for their convenient administration and reliable estimation of the three attentional networks: alerting, orienting, and executive control [42–47]. In this study, it was hypothesized that soccer players would (1) exhibit faster reaction time (RT) in milliseconds (ms) across all three attention networks, (2) demonstrate more accurate performance when faced with incongruent flanker information, and (3) demonstrate less variability in performance compared to track and field players.

## Method

### Participants

From June 2019 to January 2020, varsity athletes were recruited at pre-season baseline testing as part of a larger study that included athletes in soccer, football, hockey, basketball, volleyball, tennis, track and field, cross-country, or wrestling from York University, Toronto, Canada. All participants were varsity level players, meaning they competed at an intermediate level and possessed moderate skills and professionalism relative to their age and academic background. The current study included athletes between 17 and 24 years of age who were either playing soccer (n = 50; 50% female) or track and field (n = 50; 57% female). Participants were excluded from analyses if their mean RTs on the ANT-I were greater than three standard deviations (SDs) from the mean and if their accuracy rates were lower than 80% (ie., three track and field players were removed; n_track and field_ = 47; N = 97) [48]. No participants were actively recovering from a concussion at the time of data collection. All participants provided informed written consent following procedures approved by the Human Participants Review Sub-Committee of York University’s Ethics Review Board.

### Measures

**Attention Network Test-Interaction (ANT-I).** The ANT-I is a 10-minute computerized test measuring aspects of attention and executive control by examining RT performance across three domains: alerting (auditory tone), orienting (visual cue), and executive control (congruent and incongruent flankers) [49, 50]. Alerting refers to the ability to achieve and maintain response readiness to environmental cues and can be measured by comparing trials without or with auditory warnings before the target. Alerts are typically associated with decreased RT and increased error rate [41, 51–53]. Orienting is a function of selective attention allocation to high-priority stimuli in the environment and can be measured by comparing trials with a visuospatial cue and a target that either follows the cue or is presented opposite the cue. Performance typically deteriorates in the latter case since attention must first be disengaged then reorientated back to the target [52, 54]. Executive control involves managing conflicts and dealing with interference and can be measured by comparing trials with congruent flankers surrounding the target arrows (facing the same direction as the target) to trials with incongruent flankers (facing the opposite direction). Interference from the incongruent flankers typically deteriorates performance compared to the congruent flankers [52, 55, 56].

**Fig 1:**
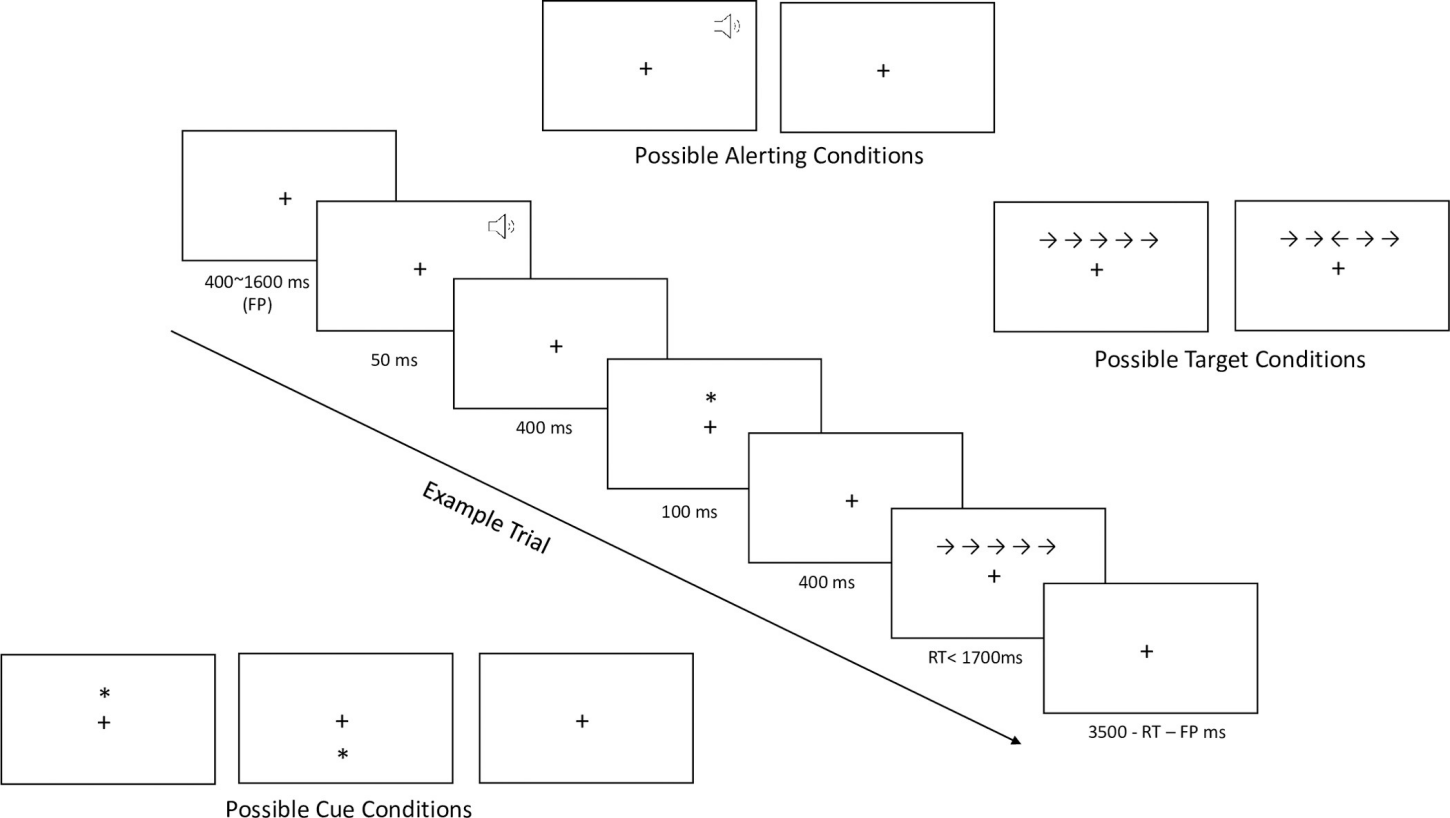
Attention Network Test (ANT-I). Example trial: Following a 400–1600 millisecond (ms) inter-trial period, the target stimulus is preceded by an alerting tone (second top panel) and a valid orienting cue (fourth top panel). The target (center) arrow is surrounded by incongruent flankers (second bottom panel). Possible cue, target (i.e., congruent and incongruent flankers), and alerting conditions are also displayed.

In our study, participants are presented with a series of five arrows located either above or below a central fixation cross and have to indicate the direction of the center arrow by pressing the "/" (right) or "z" (left) keys on a standard computer QWERTY keyboard. The test was composed of 25 practice trials and 144 test trials. The test trials contained a visual stimulus component in the form of a series of five arrows, with the center arrow pointing equally to the left or right, while the four surrounding arrows pointed in the same direction of the center arrow (congruent) in half of the trials and pointed in the opposite direction of the center arrow (incongruent) in the remaining half. The trials were presented at variable intervals, with a fixation cross appearing in the center of the screen between each trial. The intervals ranged from 400 to 1600 ms, and each trial lasted for 4450 ms. An alerting stimulus in the form of a 2000 Hz tone was played on half of the trials for 50 ms, followed by an orienting stimulus that appeared after 100 ms in the form of an asterisk on two-thirds of the trials for 50 ms. In addition, 48 trials were randomly presented with a valid spatial cue, with the asterisk located either above or below the central fixation cross, correctly signalling the upcoming position of the target stimulus. Another 48 trials were presented with an invalid spatial cue, incorrectly signalling the target stimulus’ upcoming position, and the remaining 48 trials had no cue associated with them (see Fig 1).

### Statistical analysis

Mean RT (ms) was calculated for each of the twelve combinations of independent variables and IIV was measured by calculating individual standard deviations (ISD) across all trials for each participant [52, 57, 58]. In order to examine performance across all conditions, twelve condition scores were calculated based on every possible combination of alerting (tone vs. no tone), orienting (valid vs. invalid vs. no cue) and executive control (congruent vs. incongruent flankers) factors. To examine the effect of sport type on athletes’ performance on the three attention networks, a 2 (alerting) x 3 (orienting) x 2 (executive) x 2 (sport type) repeated measures ANOVA was conducted, and findings were followed up with independent-samples t-tests. Pairwise comparisons were conducted with adjusted p-values using the Bonferroni correction. A series of independent-samples t-tests were also conducted to compare inter-group response accuracy rates as well as IIV across all trials. A Mann-Whitney U test was used to examine between-group differences in lifetime concussion history. Two-tailed alpha was set at .05 for all procedures, and data were analyzed using Statistical Package for the Social Sciences (SPSS) version 26 (IBM corp. Released, 2019). Suggested standard effect size magnitude for ηp2 are: small = 0.01; medium = 0.06; large = 0.14; and for Cohen’s d are: small = 0.20; medium = 0.50; large = 0.80 [59].

## Results

There was no significant difference in self-reported lifetime concussion history for soccer players (M = 0.62, Mdn = 0.00; SD = 1.00) and track and field players (M = 0.38, Mdn = 0.00, SD = 0.23; U = 1028.50, Z = -1.31, p = 0.190). The results of the 2x3x2x2 ANOVA indicated a significant main effect of alerting (F(1,95) = 25.68, p < .001) with a large effect size (ηp2 = .21), a main effect of orienting (F(2,190) = 144.21, p < .001), with a large effect size (ηp2 = .60), as well as a main effect of executive control (F(1,95) = 995.67, p < .001), with a large effect size (ηp2 = .91). Pairwise comparisons for the main effect of each network condition indicated faster performance in the presence of a tone, compared to no tone (p < .001; 95% CIs[-14.53, -6.35]), valid cue compared to an invalid cue (p < .001; 95% CIs[-54.65, -34.28]), or no cue (p < .001; 95% CIs[-70.06, -54.23]); and invalid cue compared to no cue (p < .001; 95% CIs[-27.01, -8.34]) across both groups. In addition, performance was faster when the flankers were congruent compared to incongruent (p < .001; 95% CIs[-109.37, -96.43]) across both groups. There was also a main effect of sport type (F(1,95) = 3.94, p = .049), with a small effect size (ηp2 = .04), with overall faster performance among soccer players (M = 597.37; SD = 65.25), compared to track and field players (M = 624.15; SD = 67.54; p = .049; d = .40), with a small-to-medium effect size.

No significant interactions were observed between sport type and the alerting (F(1, 95) = 3.43, p = .067, ηp2 = .04), orienting (F(2,190) = 1.47, p = .233, ηp2 = .02), or executive control networks (F(1,95) = 3.80, p = .054, ηp2 = .04). For exploratory purposes, post-hoc analyses were conducted. Compared to track and field players, faster RTs (ms) were observed for soccer players under alerting no tone (t(95) = -2.22, p = .029, d = .45), orienting valid cue (t(95) = -2.39, p = .019, d = .49), and executive incongruent flanker conditions (t(95) = -2.19, p = .031, d = .45; see Table 1), all with small-to-medium effect sizes. The results also indicated a small-to-medium significant difference in overall response accuracy rates between the two sports (t(95) = 2.35, p = .021, d = .48), with soccer players responding somewhat more accurately to test conditions in comparison to track and field players (97.7% versus 96.2%). Lastly, there was no statistically significant between-group difference in IIV, (t(95) = -1.75, p = .083, d = .36).

**Table 1:** Descriptive statistics and ANT-I performance by sport type, N = 97.

	Soccer (n = 50)	Track & Field (n = 47)	*p* (Cohen’s *d)*
Sex (female) *n* (%)	25 (50.00)	27 (57.45)	-
Age *M* (*SD*)	19.54 (1.55)	20.43 (1.70)	-
Concussion History *Md(*Range)	0 (0–4)	0 (0–3)	.190 (.22)
ANT-I Network Conditions (ms) *M*(*SD*)			
Alerting- Tone	594.06 (65.28)	617.02 (67.55)	.092 (.35)
Alerting- No Tone	600.68 (66.51)	631.28 (69.31)	.**029 (.45)**
Orienting- Valid Cue	558.80 (66.20)	591.63 (69.17)	.**019 (.49)**
Orienting- No Cue	623.60 (68.37)	651.14 (73.82)	.060 (.39)
Orienting- Invalid Cue	609.70 (70.53)	629.67 (70.40)	.166 (.28)
Executive- Congruent Flanker	549.10 (63.30)	569.52 (59.52)	.105 (.33)
Executive- Incongruent Flanker	645.64 (68.96)	678.78 (79.89)	.**031 (.45)**
Overall RT (ms) *M*(*SD*)	597.37 (65.25)	624.15 (67.54)	**.050 (.40)**
Accuracy Percentage *M*(*SD*)	97.74 (2.15)	96.23 (3.93)	.**021 (.48**)
IIV (ISD) *M*(*SD*)	6.45 (1.94)	7.35 (3.03)	.083 (.36)

*Note*. Data only includes participants who have achieved an 80% accuracy rate on ANT-I Abbreviations: RT = reaction time; IIV = intra-individual variability; ISD = individual standard deviation; ms = milliseconds

Bolded text indicates *p*<0.05.

## Discussion

The current study examined speed and stability of performance on a measure of attention and executive control among strategic (i.e., soccer) and static sport varsity athletes (i.e., track and field). It was hypothesized soccer players would (1) exhibit faster RT across all three attention networks, (2) demonstrate better performance when faced with incongruent flanker information, and (3) demonstrate less variability in performance compared to track and field players. Consistent with prior studies examining ANT-I performance, both strategic and static players displayed faster performance in conditions where there was a tone compared to no tone, a valid cue compared to an invalid cue or no cue, and where the flankers were congruent compared to incongruent [49, 50]. Consistent with our first hypothesis, a small difference in overall RT between groups was observed, with strategic athletes demonstrating faster overall performance compared to static athletes. Previous research examining cognitive performance between athletes in strategic and non-strategic sports (i.e., static or interceptive sports) has revealed mixed results depending on the aspects of cognition that were measured, how that domain was measured (i.e., via computerized tests of cognition or paper and pencil neuropsychological measures), as well as which groups were examined (i.e., elite athletes, disabled athletes, single sport, or mixed sports athletes) [14, 15, 18, 19, 22, 23].

To date, there is some evidence of superior performance (i.e., faster RTs and higher accuracy) on tasks of motor speed, visual attention, and inhibition among athletes participating in strategic sports [15, 18, 19, 23]. However, others have reported that elite athletes participating in endurance (i.e., marathon runners) or motorically complex (i.e., Wushu) sports did not demonstrate significantly different performance on neuropsychological tests of inhibition, cognitive flexibility, and planning (i.e., Stroop; Wisconsin Card Sorting Test; WCST, Tower of London) [28]. Similarly, no differences were observed between externally-paced and self-paced athletes on similar measures of inhibition and cognitive flexibility (i.e., Stroop; D-KEFS Tower Test) [30]. In the present study, using athletes who were comparable in age, years of education, and level of play (i.e., varsity), we observed evidence of overall faster RTs in strategic sport athletes. While this difference was relatively small (i.e., in the order of 26 ms), the small effects are meaningful and significant [60]. It is possible that a difference of a few milliseconds in competitive sports can not only protect athletes from severe injuries [61, 62] but also place them at an advantage when processing multiple external stimuli, engaging in coordinated actions and executing complex motor functions [63, 64].

Further examination of performance across differing conditions revealed that soccer players were less affected under more difficult alerting (i.e., faster RTs in the absence of a tone) and executive control conditions (i.e., faster RTs with incongruent flankers), which was consistent with our second hypothesis. This suggests these athletes were able to maintain their speed despite the need for increased vigilance, error detection, and conflict resolution compared to track and field players. Performance variability across trials for the two groups was examined and a statistically significant difference was not observed, which was inconsistent with our third hypothesis. However, it is possible for an effect to be observed with a larger and more diverse sample. Playing strategic sports often necessitates the use of enhanced inhibitory functions in order to tune out irrelevant and distracting environmental stimuli and to attend to informative cues regarding each player’s position [65, 66]. Therefore, it is possible the specific cognitive demands of playing a strategic sport, such as soccer, may train athletes to perform better on domain-general tasks of attention and executive control. Recent findings of a study using the Cambridge Brain Sciences (CBS) test battery support this claim by indicating that young athletes playing team sports (i.e., soccer, basketball, volleyball, korfball, or hockey) display enhanced executive function in contrast to athletes playing self-paced sports (i.e., cycling, swimming, or athletics) and sedentary controls [67]. This would align with the broad cognitive skill transfer hypothesis, which suggests that training in one cognitive task enhances performance on another related, yet untrained cognitive task [13, 25–27]. However, given the cross-sectional nature of these data, it is difficult to determine whether the observed differences are due to self-selection (i.e., whether those with superior attention/executive control choose to engage in strategic sports) as opposed to the effects of specific training. This remains a key question for future research to determine the role of domain-general capacities in highly skilled performance.

It was also observed that soccer players responded more accurately on the ANT-I compared to track and field players, although this difference was relatively small (97.74% versus 96.23%, respectively). Nevertheless, a 1.5% difference in accuracy could have a significant effect on performance outcomes for an athlete, particularly in groups at this level of skill [60]. Prior research has reported that athletes in open-skilled sports (i.e., volleyball) perform more accurately than athletes from closed-skilled sport and non-sport controls on measures of selective information processing [23]. Therefore, it is possible that athletes playing strategic sports may have an advantage in information processing efficiency, both in terms of speed and accuracy, which may translate to maximizing top-down processes under time pressure in anticipation of the opponents’ tactical approaches, as well as the ball’s movements across the field [13, 21, 23, 68, 69]. Future research is needed to further understand this relationship. These characteristics are also referred to as "game intelligence", which allow strategic athletes to make fast and effective decisions based on their prior training [8, 70]. On the one hand, the observed findings of increased speed and accuracy may reflect cognitive adaptability that soccer players develop over time as they learn to adjust to their opponents’ advances in highly variable circumstances. On the other, the findings may reflect selection by athletes (i.e., self-selection) or coaches (i.e., through talent selection initiatives) of those who have predispositions to success in these activities.

Overall, this study contributes to the growing body of literature examining associations between sport type and performance on domain-general cognitive tasks in athletes. It highlights the potential bidirectional relationship between cognitive benefits and engaging in strategic, open-skill, invasion-style activities in young adulthood. Nevertheless, there are several limitations associated with the interpretations that require addressing. First, this study did not include objective measures of cardiovascular fitness or any anthropometric variables such as weight, height, and body mass index, which may have contributed to individual differences. Future studies would benefit from incorporating heartrate monitors and other anthropometric and physiological indices—such as percentage of body fat and muscle mass—to address whether a superior fitness status or long-term engagement in athletic training is associated with differences in cognitive performance. A further limitation of this study is that it did not account for behavioural and socioemotional functions (e.g., mood, motivation, sleep habits), as well as overall neuromuscular fitness (e.g., postural control, muscle strength, agility, muscle reflex activity, etc.), which may have been associated with improved performance on the cognitive test. Future studies would benefit from including these factors in their analyses for a more thorough characterization of the processes by which sports participation influences cognitive performance. Lastly, characterizing athletes’ years of training and specialization of training (e.g., single sport versus multi-sport participation) would help elucidate the association between sport engagement and cognitive functioning.

## Conclusions

This study found that, in a sample of varsity athletes of comparable education, sex, and level of play, those engaging in strategic sports demonstrated increased speed, accuracy, and conflict resolution compared to static athletes on a non-sport-specific cognitive test. Findings from this study align with prior research suggesting that sport type may be associated with differential cognitive profiles [14–22] and suggests that the deliberate practice of strategic sports may play a role in enhancing performance on non-sport-specific cognitive tasks. However, it is notable that the observed differences were relatively small (effect sizes less than 0.50), suggesting that differences in cognitive performance may be subtle when using relatively a well-matched sample of highly fit individuals, or that differences may be explained by a disproportionate representation of athletes who are predisposed to succeed in these tasks as a result of selection bias. Future research should explore the possible influence of different levels and specialization of training on attention and executive control performance. In addition, further research examining relationships between domain-specific athletic training and cognitive performance on a variety of sport-and non-sport-related test batteries is warranted.
